# Supplementary material for: A single cell RNA sequence atlas of the early Drosophila larval eye
Source: BMC Genomics. 2024 Jun 19;25:616. doi: 10.1186/s12864-024-10423-x (PMC11186242; doi:10.1186/s12864-024-10423-x)

Supplemental Figure 1

A Early Larval Eye Cell Numbers

Cell Type	Expected #	Expected %	Observed #	Observed %
AUnd+MF	2000	65	2,117	80
PUnd	750	21	254	10
R8	125	4	79	3
Rcells	500	10	191	7
**Total**	**4625**	**100**	**2,641**	**100**

B Mid-Larval Eye Cell Numbers

Cell Type	Expected #	Expected %	Observed #	Observed %
AUnd+MF	3000	42	3,132	39
PUnd+SMW	1650	23	3077	38
R8	450	7	431	6
R2/5	450	7	528	6
R3/4	400	**6**	559	7
R1/6	350	**5**	152	2
R7	350	**5**	86	1
Cones	350	**5**	136	1
**Total**	**7000**	**100**	**8101**	**100**

C Combined Eye Cell Numbers

Cell Type	Observed #
AUnd+MF	5,447
PUnd+SMW	3,727
R8	487
R2/5	796
R3/4	586
R1/6	159
R7	98
Cones	156
**Total**	**11,546**

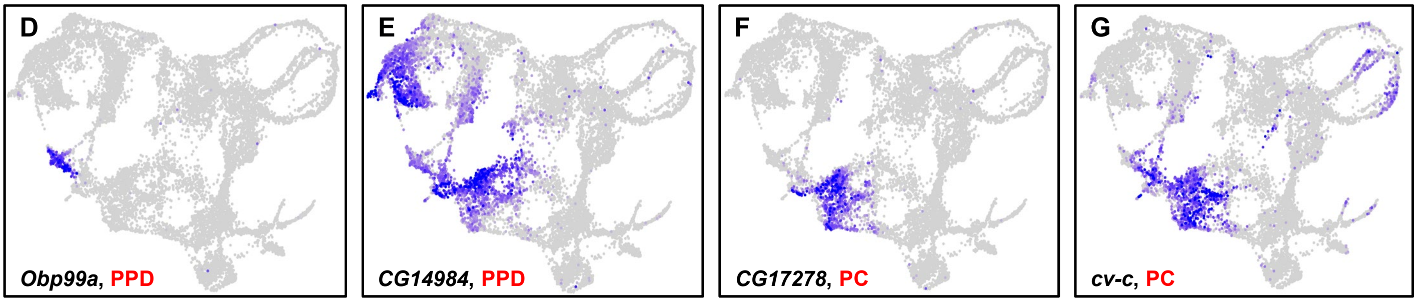

Supplemental Figure 2

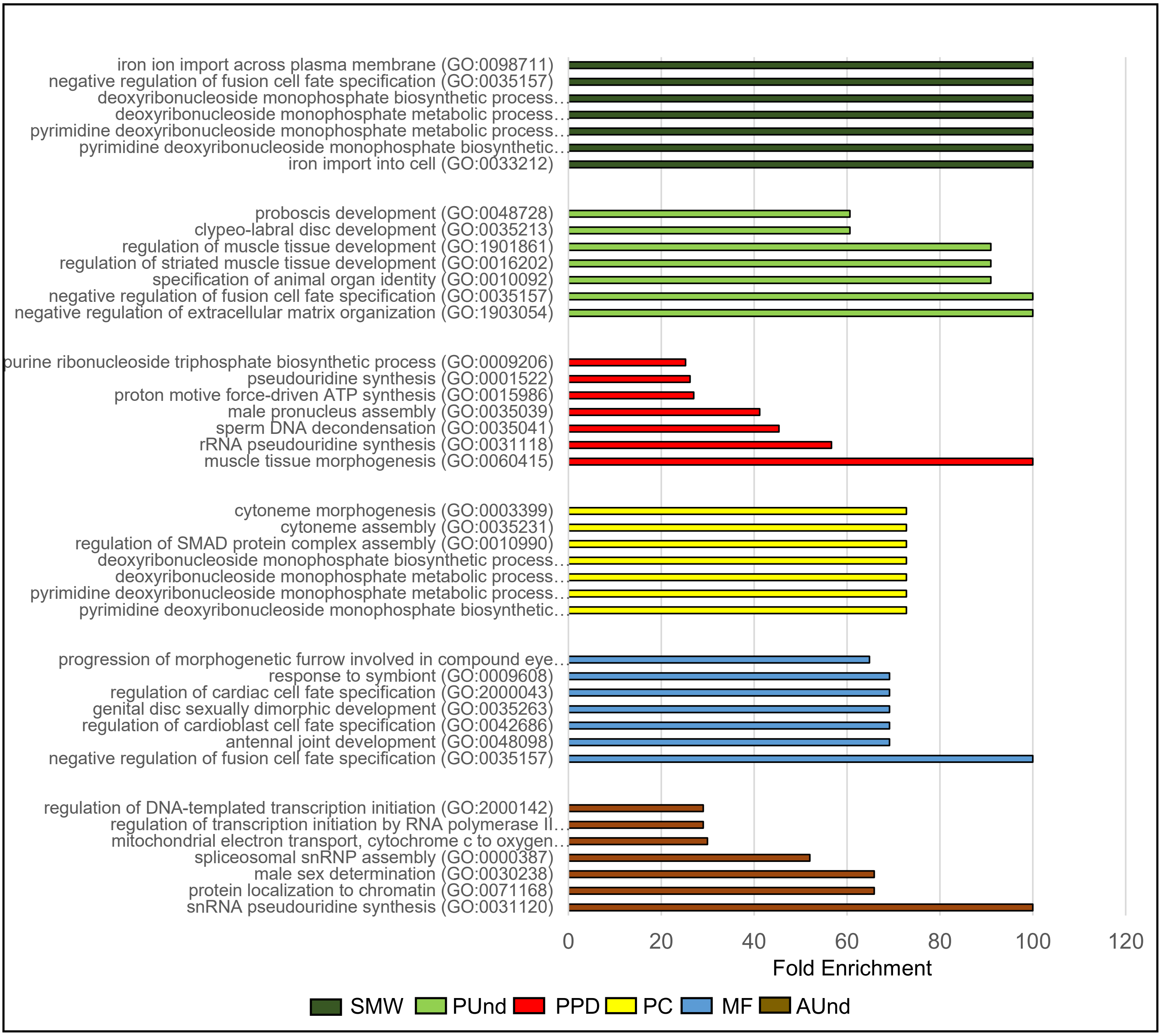

Supplement: Supplementary file 1 — Supplementary Material 1. [file 12864_2024_10423_MOESM1_ESM.pdf]
